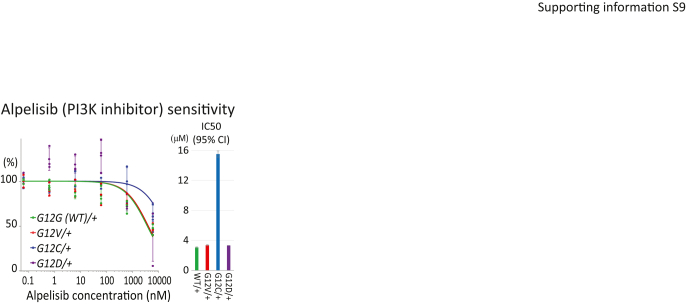# Endogenous oncogenic KRAS expression increases cell proliferation and motility in near-diploid hTERT RPE-1 cells

**DOI:** 10.1016/j.jbc.2024.107409

**Published:** 2024-05-23

**Authors:** Naushin L. Hindul, Lauren R. Abbott, Sumaya M.D. Adan, Kornelis R. Straatman, Andrew M. Fry, Kouji Hirota, Kayoko Tanaka

**Affiliations:** 1 Department of Molecular and Cell Biology, University of Leicester, Leicester, UK; 2 Advanced Imaging Facility, University of Leicester, Leicester, UK; 3 Department of Chemistry, Graduate School of Science, Tokyo Metropolitan University, Hachioji, Japan

**Keywords:** small GTPase, focal adhesion, cell cycle, cell motility, cell proliferation, cell signaling

## Abstract

About 18% of all human cancers carry a mutation in the *KRAS* gene making it among the most sought-after anticancer targets. However, mutant KRas protein has proved remarkably undruggable. The recent approval of the first generation of RAS inhibitors therefore marks a seminal milestone in the history of cancer research. It also raises the predictable challenges of limited drug efficacies and acquired resistance. Hence, new approaches that improve our understanding of the tumorigenic mechanisms of oncogenic RAS within more physiological settings continue to be essential. Here, we have used the near-diploid hTERT RPE-1 cells to generate isogenic cell lines in which one of the endogenous *KRAS* alleles carries an oncogenic *KRAS* mutation at glycine 12. Cells with a *KRAS*^*G12V/+*^, *KRAS*^*G12C/+*^, or *KRAS*^*G12D/+*^ genotype, together with WT *KRAS*^*G12G(WT)/+*^ cells, reveal that oncogenic *KRAS.G12X* mutations increase cell proliferation rate and cell motility and reduced focal adhesions in *KRAS*^*G12V/+*^ cells. Epidermal growth factor -induced phosphorylation of ERK and AKT was comparable between *KRAS*^*G12V/+*^, *KRAS*^*G12C/+*^, *KRAS*^*G12D/+*^, and *KRAS*^*G12G(WT)/+*^ cells. Interestingly, *KRAS*^*G12X/+*^ cells showed varying responses to distinct inhibitors with the *KRAS*^*G12V/+*^ and *KRAS*^*G12D/+*^ cells more sensitive to hydroxyurea and MEK inhibitors, U0126 and trametinib, but more resistant to PI3K inhibitor, PIK-90, than the *KRAS*^*G12G(WT)/+*^ cells. A combination of low doses of hydroxyurea and U0126 showed an additive inhibition on growth rate that was greater in *KRAS*^*G12V/+*^ than WT cells. Collectively, these cell lines will be a valuable resource for studying oncogenic RAS signaling and developing effective anti-KRAS reagents with minimum cytotoxicity on WT cells.

The RAS family of small GTPases acts as a signaling hub, regulating fundamental biological activities through multiple downstream pathways (1). Human *RAS* genes are frequently mutated in cancers, and the germline mutations cause disorders termed RASopathies, underlining their physiological importance (2, 3). Among the three human *RAS* genes, *KRAS*, *NRAS*, and *HRAS*, the *KRAS* gene exhibits the highest mutation rate, reaching about 18% across all human cancers (Catalogue of Somatic Mutations in Cancer (COSMIC), v98). Over 80% of the *KRAS* oncogenic mutations are missense mutations at glycine position 12, which is most often mutated to aspartic acid (G12D), valine (G12V), or cysteine (G12C) (4).

Once described as “undruggable,” the oncogenic KRas protein can today be targeted on account of the extensive efforts inspired by the first KRas.G12C inhibitor developed by Shokat *et al.* (5). The KRas.G12C inhibitors are now available for use in the clinic (6, 7), while promising KRas.G12D, KRas.G12V, and pan-Ras inhibitors have also been developed (8, 9, 10, 11). However, there remain significant pending challenges, not least acquired resistance to these inhibitors (12, 13). To design rational and effective counteracting strategies, it is crucial to obtain deeper insights on how oncogenic KRas signaling influences cell processes.

Early studies that relied on *RAS* overexpression in cultured cells revealed the high potency of oncogenic Ras proteins to cause various cancer-relevant phenotypes, including hyperactivation of downstream signaling components (such as extracellular signal-regulated kinase [ERK] and AKT), cell transformation, cell migration, and senescence (14). However, through the elegant analysis of *KRAS* mouse models, the importance of looking at endogenous *KRAS* oncogenic mutations has become well-recognized (14, 15, 16, 17, 18). A simpler and cost-effective alternative to mouse models may be examining the consequences of expression of endogenous *KRAS* mutations in human cultured cells derived from normal tissues and immortalized through the expression of human telomere reverse transcriptase (hTERT). This would allow phenotypic changes to be determined in cells that retain a stable and near-diploid karyotype (19, 20). Previously, hTERT-immortalized HME1 (mammary epithelial) cells expressing a *KRAS.G13D* mutant and hTERT-IMEC (mammary epithelial) cells expressing a *KRAS.G12V* mutant were generated using adeno-associated viral-mediated gene targeting (21, 22). These studies established that these endogenous oncogenic *KRAS* mutations do not cause cellular transformation, unlike their overexpression (21, 22). The studies also reported that the cell proliferation rate (21) or morphology (22) of the cells carrying these endogenous oncogenic *KRAS* mutations were comparable to the parental cells.

Here, we report the generation of isogenic human cell lines carrying heterozygous oncogenic *KRAS* mutations using hTERT-immortalized retinal pigment epithelial (RPE-1) cells, one of the most well-characterized hTERT-immortalized cell lines originated from normal human tissue. However, as the original hTERT RPE-1 cell line (CRL4000, American Type Culture Collection (ATCC)) turned out to carry the c.30_35dup *KRAS* mutation at one of the *KRAS* loci, we targeted this locus so that it was converted into the oncogenic *KRAS.G12V*, *KRAS.G12D*, or *KRAS.G12C* mutations, as well as generating a WT *KRAS.G12G.* These will be referred here as *KRAS*^*G12V/+*^, *KRAS*^*G12D/+*^, *KRAS*^*G12C/+*^, and *KRAS*^*G12G(WT)/+*^ cell lines.

Characterization of these isogenic cell lines revealed that all three endogenous oncogenic *G12X* mutations resulted in increased cell proliferation and cell motility compared to the G12G (WT) counterpart. Further analyses of the *KRAS*^*G12X/+*^ cell lines showed that the epidermal growth factor (EGF)-stimulated ERK and AKT activation profiles were comparable to the *KRAS*^*G12G(WT)/+*^ cells. Interestingly, compared to the *KRAS*^*G12G(WT)/+*^ cells, the *KRAS*^*G12V/+*^ cells were more sensitive to hydroxyurea (HU) and MEK inhibitors, U0126 and trametinib, but more resistant to the PI3K inhibitor, PIK-90. The results highlight the impact of a single endogenous oncogenic *KRAS* mutation and showcase the value of these cell lines as a powerful tool in the study of oncogenic *KRAS*-induced pathogenesis.

## Results

### Identification of a heterozygous c30_35dup mutation in *KRAS* in the hTERT RPE-1 cell line

**Supplemental Figure S1:**
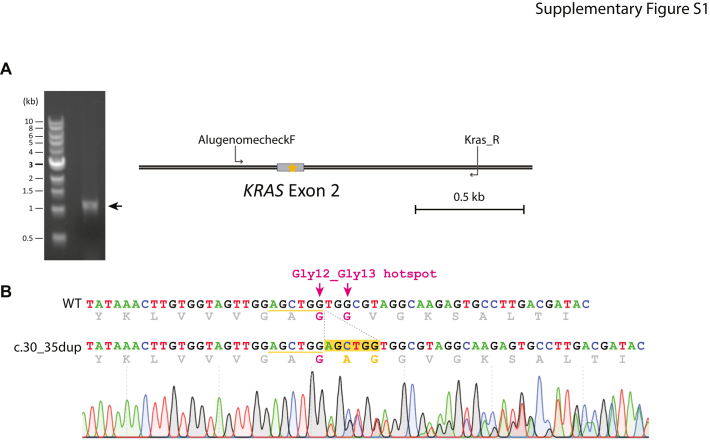


During the process of establishing a protocol to genotype exon 2 of the *KRAS* gene locus, we found that one of the *KRAS* alleles has the insertion mutation, termed c.30_35dup. As our lab stock of hTERT RPE-1 cells had been obtained more than 10 years ago, we purchased a fresh aliquot of hTERT RPE-1 cells (CRL-4000) from ATCC in January 2021. However, we found that this too contained the heterozygous c.30_35dup mutation (Fig. S1*A*). The presence of this mutation in these cells has been previously reported (21, 23), indicating that the original hTERT RPE-1 cell line likely carries the c.30_35dup mutation in one of the *KRAS* alleles. The c.30_35dup mutation causes the duplication of Ala11_Gly12 (p.Ala11_Gly12dup mutation), resulting in the insertion of two additional amino acids, Ala-Gly, at positions 13 and 14 (Fig. S1*B*). Considering that Gly12 and Gly13 are hotspots for the *KRAS* oncogenic mutation, the c.30_35dup mutation is highly likely to affect KRas function. In fact, in the Catalogue of Somatic Mutations in Cancer (COSMIC v.98), 6 cases of the c.30_35dup mutation are reported out of 49,887 cases of *KRAS* mutation. Furthermore, enrichment of the active GTP-bound form of KRas in lysates of the hTERT RPE-1 cell line was reported (21). Therefore, in this study, we decided to target the chromosome carrying the c.30_35 *KRAS* mutation to generate isogenic cell lines of *KRAS*^*G12G*^ ^*(WT)/+*^, *KRAS*^*G12V/+*^, *KRAS*^*G12C/+*^, and *KRAS*^*G12D/+*^.

### Integration of *KRAS*^*G12G/C/D/V*^ at the endogenous *KRAS* locus in hTERT RPE1 cells

**Figure 1:**
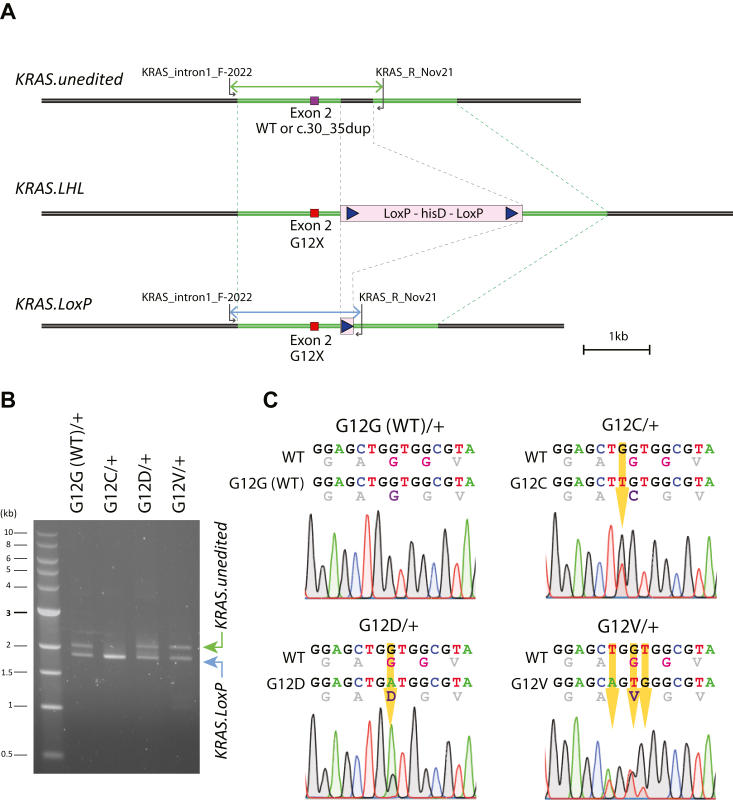
**Successful removal of the *KRAS.c.30_35dup* allele to generate isogenic *KRAS***^***G12X/+***^**cell lines.***A*, a schematic diagram of chromosome editing of the *KRAS* gene locus. The original unedited *KRAS* locus is shown as *KRAS.unedited*. The parental cell line, hTERT RPE-1, carries one WT *KRAS* and one *c.30_35dup* mutant *KRAS* allele. A DNA fragment encoding histidinol dehydrogenase (hisD), flanked by LoxP sites (LoxP-HisD-LoxP, LHL) together with the homologous arms (highlighted in *green*) was inserted into intron 2 of the *KRAS* gene to create the *kras.LHL* locus. During this process, the c.30_35dup allele can be removed and replaced with the intended *KRAS.G12X* alleles. The generated cells were further treated with doxycycline and 4-OHT so that the LHL cassette was excised, generating the *KRAS.LoxP* allele. A pair of primers, KRAS_intron1_F-2022 and KRAS_R_Nov21, are indicated in the diagram. *B*, PCR-based genotyping. The PCR reaction was conducted using primers KRAS_intron1_F-2022 and KRAS_R_Nov21. The primer KRAS_intron1_F-2022 is outside of the fragment used for *KRAS* gene editing. The PCR reaction produced the expected products of 2163 bp for *kras.unedited* and 1908 bp for *kras.LoxP*. For the *KRAS*^*G12C/+*^ cells, both chromosomes carry the *KRAS.LoxP* allele, and the PCR product shows only one band of 1908 bp. However, only one of them has the *KRAS.G12C* mutation, as shown in (*C*). *C*, sequencing of the genome PCR products confirms the successful introduction of the intended G12X mutations. The PCR products presented in (*B*) were sequenced to confirm the successful replacement of the c.30_35dup mutation with the intended G12X alleles. Below the nucleotide sequences, corresponding amino acid sequences are shown in *gray*. The Gly12-Gly13 oncogenic mutation hotspot is indicated in *magenta*, and the position 12 amino acids are indicated in *purple*. 4-OHT, 4-hydroxy tamoxifen; hTERT, human telomere reverse transcriptase; LHL, LoxP-hisD-LoxP; RPE, retinal pigment epithelial.

**Supplemental Figure S2:**
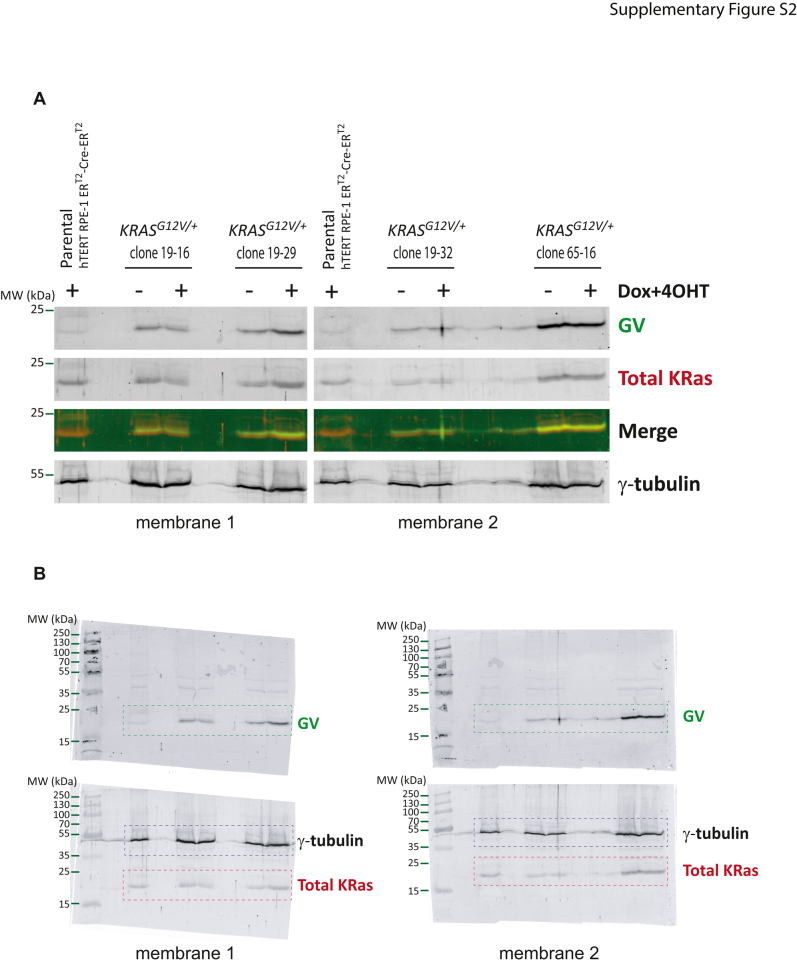


The *KRAS* gene locus was edited through CRISPR-Cas9 mediated recombination of a DNA fragment spanning the *KRAS* exon 2 that encodes Gly12 (Fig. 1*A*). We used the ER^T2^-Cre-ER^T2^ hTERT RPE-1 cells that we had previously generated (24) to facilitate the removal of the selection marker (histidinol dehydrogenase, hisD), which is sandwiched between two LoxP sites (Fig. 1*A*, LoxP-hisD-LoxP, or LHL). In this cell line, the expression and activation of the ER^T2^-Cre-ER^T2^ can be induced by the addition of doxycycline and 4-hydroxy tamoxifen (4-OHT) to the culture media, leading to removal of the LHL cassette. The *KRAS* allele was targeted by a CRISPR-Cas9 construct, pX330-kras_CRISPR, and the c.30_35dup mutation was replaced with the G12V, G12D, G12C or G12G (WT) alleles using the pKH-His-DA-Ap plasmid, as previously described (24). We originally intended to generate KRas.G12X inducible cell lines where the presence of the LHL cassette represses the expression of the KRas.G12X. However, when the KRas.G12V expression was examined using an anti-G12V specific antibody, the LHL cassette turned out not to repress the KRas.G12V expression. Cells harboring the *KRAS.LHL* allele, retaining the LHL cassette, were found to express a similar level of the G12V variant compared to cells harboring the *KRAS.LoxP* allele from which the LHL cassette had been removed (Fig. S2). Therefore, we decided to analyze the phenotypes resulting from constitutive expression of endogenous oncogenic *KRAS* mutant, and removed the LHL cassette so that the genomic sequence of the two *KRAS* loci were more comparable (Fig. 1, *A* and *B*).

We successfully generated one clone of G12G (WT), four clones of G12V (19–16, 19–29, 19–32, and 65–16), one clone of G12C and one clone of G12D that carry the desired mutations at exon 2 in a heterozygous manner (Fig. 1*B*). In the following analyses, one of the four G12V clones (clone 19–16) and each clone of the other genotypes were analyzed. Sequencing confirmed that the *KRAS* coding sequences of these clones did not contain additional mutations (Fig. S3, *A–E*). For amplification of the *KRAS* coding sequences for sequencing analysis, we used a pair of primers that can amplify both *KRAS4A* and *KRAS4B*, two *KRAS* splice variants. In all cell lines, the sequence signals were dominated by the *KRAS4B* transcript, and the *KRAS4A* transcript could not be detected (Fig. S3, *A–E*). For each mutant cell line, both WT and the G12X variant sequencing signals were detected at similar intensities, confirming that all cell lines carry heterozygous *KRAS.G12X* mutations (Fig. S3, *A–E*).

### KRAS.G12X oncogenic mutations cause an increased rate of proliferation

**Figure 2:**
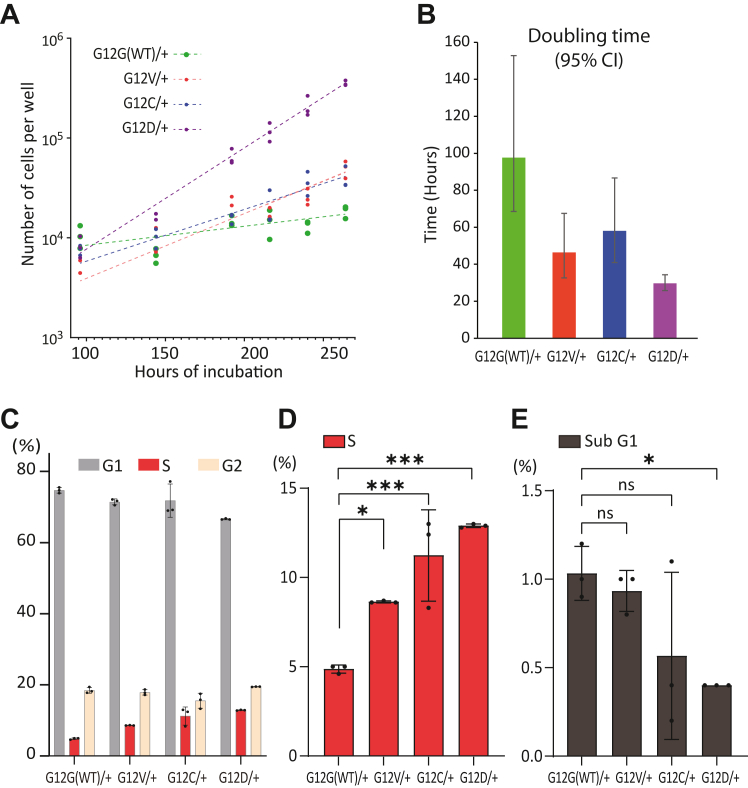
**One copy of the endogenous oncogenic *KRAS* mutation causes an increase in cell proliferation.***A*, proliferation rates of the *KRAS*^*G12X/+*^ cells. Exponential growth curves were generated by fitting to three biological replicates using Prism (GraphPad). *Y*-axis is logarithmic. *B*, oncogenic *KRAS.G12X* mutations substantially reduce the doubling time. From the data presented in (*A*), the best-fit doubling time for each cell line was estimated using Prism (GraphPad). Error bars represent a 95% confidence interval (CI). *C*, distinct cell cycle profiles between the *KRAS*^*G12X/+*^ and the *KRAS*^*G12G(WT)/+*^ cells. Flow cytometry analysis for the DNA contents was conducted for the *KRAS*^*G12X/+*^ and *KRAS*^*G12G(WT)/+*^ cells. A summary result of the biological triplicates presented in Fig. S4 is presented to show the mean and SD values of the cell cycle populations for each sample. Considering the reduced doubling time of the *KRAS*^*G12X/+*^ cells, as shown in (*B*), the G1 phase of the cell cycle is estimated to be substantially reduced in the *KRAS*^*G12X/+*^ cells. *D*, the S phase population is increased in the oncogenic *KRAS*^*G12X/+*^ cells compared to the *KRAS*^*G12G(WT)/+*^ cells. The S phase population of the flow cytometry data presented in Fig. S4 is summarized, and the mean and SD values are presented. Ordinary one-way ANOVA followed by *post hoc* Dunnnett’s multiple comparisons test showed that the *KRAS*^*G12X/+*^ samples showed significant differences from the *KRAS*^*G12G(WT)/+*^ cells. *E*, the sub-G1 population was not increased in the oncogenic *KRAS*^*G12X/+*^ cells compared to the *KRAS*^*G12G(WT)/+*^ cells. The sub-G1 population of the flow cytometry data presented in Fig. S4 is summarized, and the mean and SD values are presented. Ordinary one-way ANOVA followed by *post hoc* Dunnnett’s multiple comparisons test showed that the sub-G1 population was comparable between the *KRAS*^*G12G(WT)/+*^ and *KRAS*^*G12V/+*^ or *KRAS*^*G12C/+*^ cells and was slightly decreased in the *KRAS*^*G12D/+*^ mutant cells.

**Supplemental Figure S4:**
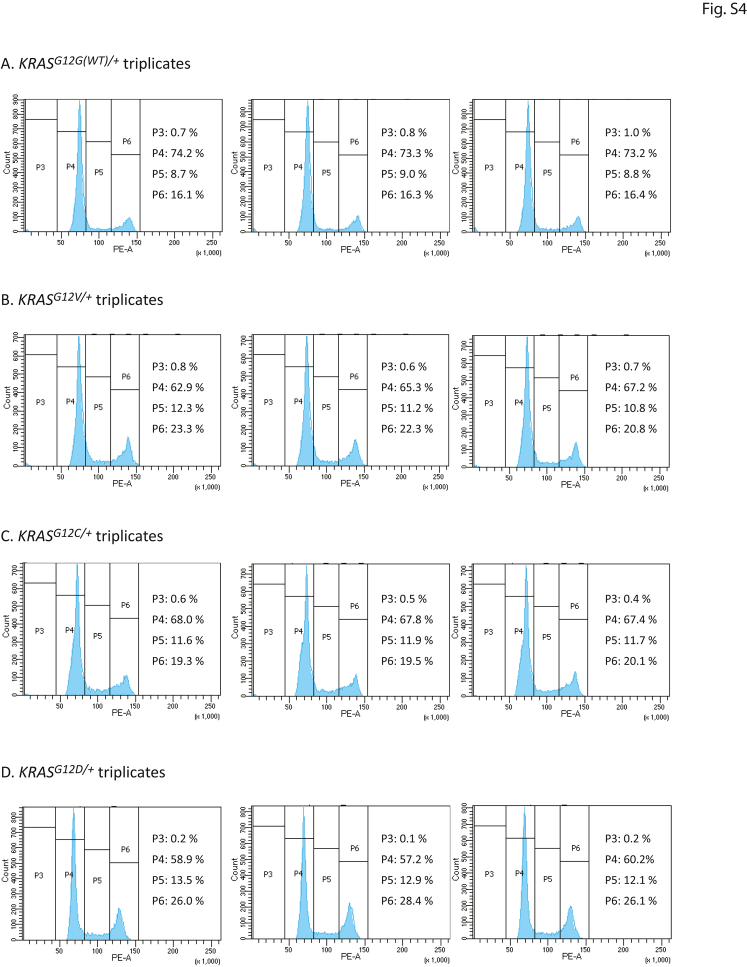


One of the critical features of cancer cells is the loss of cell cycle control that leads to hyperplasia. All the generated oncogenic *KRAS*^*G12X/+*^ mutant cells showed an increased rate of cell proliferation, with the doubling time reduced approximately two-fold compared to the control *KRAS*^*G12G(WT)/+*^ cells (Fig. 2, *A* and *B*). This demonstrates that one copy of a *KRAS.G12X* oncogenic mutation is sufficient to cause a key cancer-related phenotype. Flow cytometry analysis of the asynchronously growing oncogenic *KRAS*^*G12X/+*^ and *KRAS*^*G12G(WT)/+*^ cells showed that there was an increase in the proportion of cells in the S phase with a concomitant reduction of cells in G1 in the *KRAS*^*G12X/+*^ cell lines (Fig. 2, *C* and *D* and Fig. S4). Considering the shorter doubling times of the oncogenic *KRAS*^*G12X/+*^ cells, the times spent in S and G2 phases are estimated to be comparable between the oncogenic *KRAS*^*G12X/+*^ and *KRAS*^*G12G(WT)/+*^ cells, but the duration of the G1 phase of the cell cycle is substantially reduced in the oncogenic *KRAS*^*G12X/+*^ cells suggesting loss of restriction point control. Meanwhile, sub-G1 populations in these cell lines were comparable, except for a minor decrease in the *KRAS*^*G12C/+*^ and *KRAS*^*G12D/+*^ cells, indicating that the endogenous oncogenic *KRAS*^*G12X/+*^ mutations do not cause substantial cell death (Fig. 2*E* and Fig. S4).

### *KRAS*^*G12V/+*^ oncogenic mutation causes increased cell motility and reduced paxillin structures

**Figure 3:**
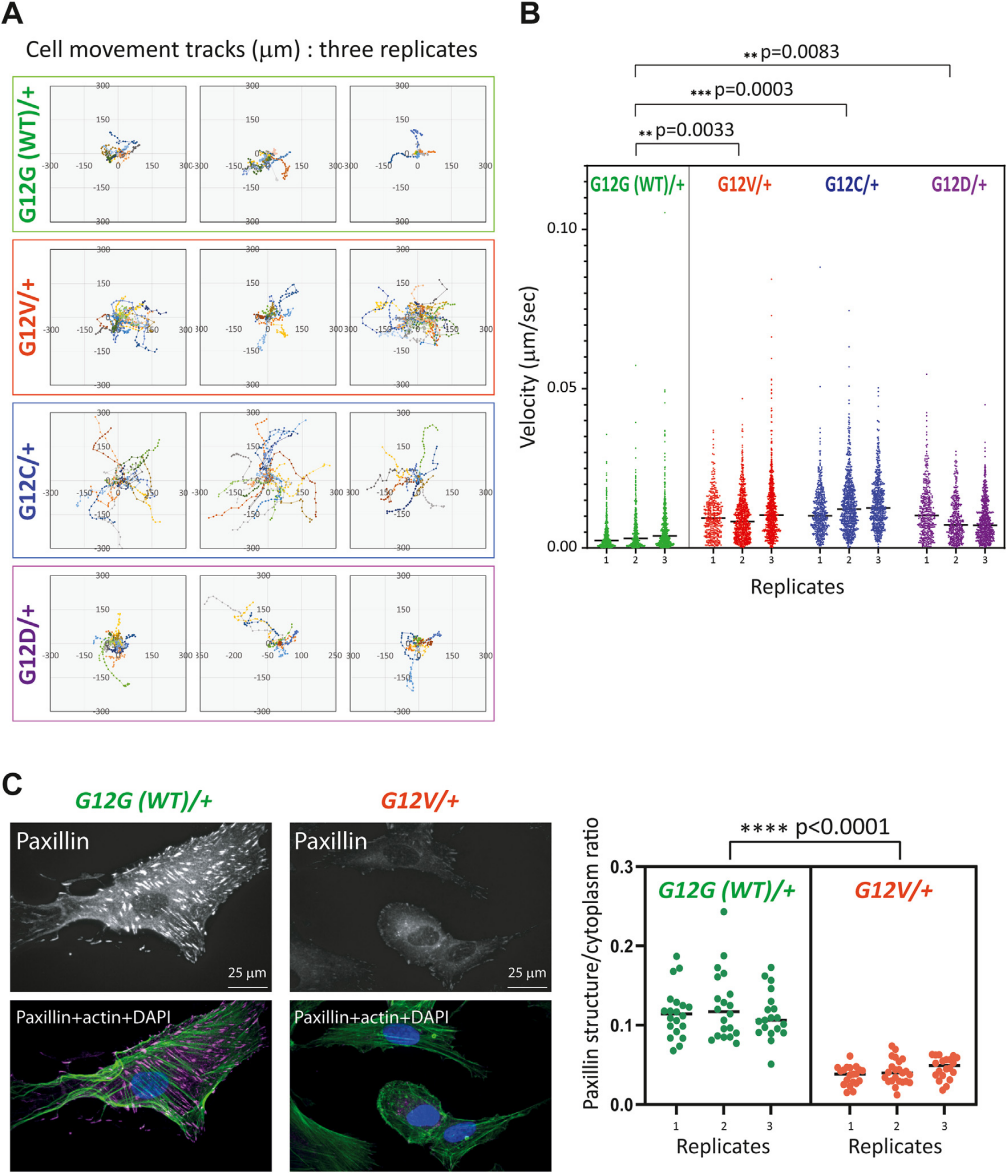
**The *KRAS***^***G12X/+***^**cells show enhanced motility.***A*, the *KRAS*^*G12X/+*^ and the *KRAS*^*G12G(WT)/+*^ cells were cultured on collagen-coated plates, and the cell movement was recorded every 20 min for 31 time frames using ptychographic phase imaging. Each cell movement was tracked and analyzed using the Livecyte system (Phasefocus) as described in the Experimental procedures. Data from three biological replicates from each cell line are presented. The positions of cell tracks are plotted on an *X*-*Y* axis, where the start of each track (time 0) is set to the center of the plot ((x, y) = (0, 0)). Each track is shown in a different color. *B*, the dataset presented in (*A*) was analyzed to deduce the velocity of each cell from one time frame to the next at every time point. The obtained values are plotted for each biological replicate and are analyzed with a nested one-way ANOVA followed by *post hoc* Dunnett’s multiple comparisons test (Prism, GraphPad), which shows an increase in the *KRAS*^*G12X/+*^ cells. *C*, a decrease in the paxillin signals in the *KRAS*^*G12V/+*^ cells. The status of paxillin structures in the *KRAS*^*G12V/+*^ and the *KRAS*^*G12G(WT)/+*^ cells were visualized by immunofluorescence microscopy as described in the Experimental procedures. The actin stress fiber and the nuclei were also counter-stained. The ratio of the area occupied by the paxillin structures and the cytoplasm was deduced for each image, as stated in Fig. S5. For each biological replicate, 20 images were analyzed, and the data from three biological replicates were analyzed by a nested *t* test (Prism, GraphPad). The area occupied by the paxillin structures is significantly reduced in the *KRAS*^*G12V/+*^ cells.

Another key phenotype of cancer cells is an increase in migration that contributes to their metastatic potential. The generation of a human HEK293 stable cell line with the expression of an *HRAS.G12V* oncogenic protein suggested that this mutation can cause an increase in cell motility (25). To examine whether the endogenous *KRAS*^*G12X/+*^ oncogenic mutation affects cell motility, live cell imaging was conducted using a label-free ptychographic imaging system (26). This allowed us to plot cell movement tracks over time revealing substantially enhanced motility for the oncogenic *KRAS*^*G12X/+*^ compared to *KRAS*^*G12G(WT)/+*^ cells (Fig. 3*A*). Quantification confirmed the increased velocity of cells carrying the oncogenic *KRAS*^*G12X/+*^ mutations (Fig. 3*B*). We pursued further analysis of the *KRAS*^*G12X/+*^ cells for the alternation in the cellular distribution of paxillin, an integral component of the focal adhesion complex. In support of this increased motility phenotype, immunofluorescence microscopy analysis indicated a significant reduction in structures that stained positive for paxillin in the *KRAS*^*G12V/+*^ cells (Fig. 3*C*). These data suggest that one copy of the endogenous *KRAS.G12X* oncogenic mutation is sufficient to enhance cell motility and potentially alter cell adhesion properties.

### EGF-stimulated ERK and AKT signaling is comparable in *KRAS*^*G12X/+*^*oncogenic* mutants and *KRAS*^*G12G(WT)/+*^ cells

**Figure 4:**
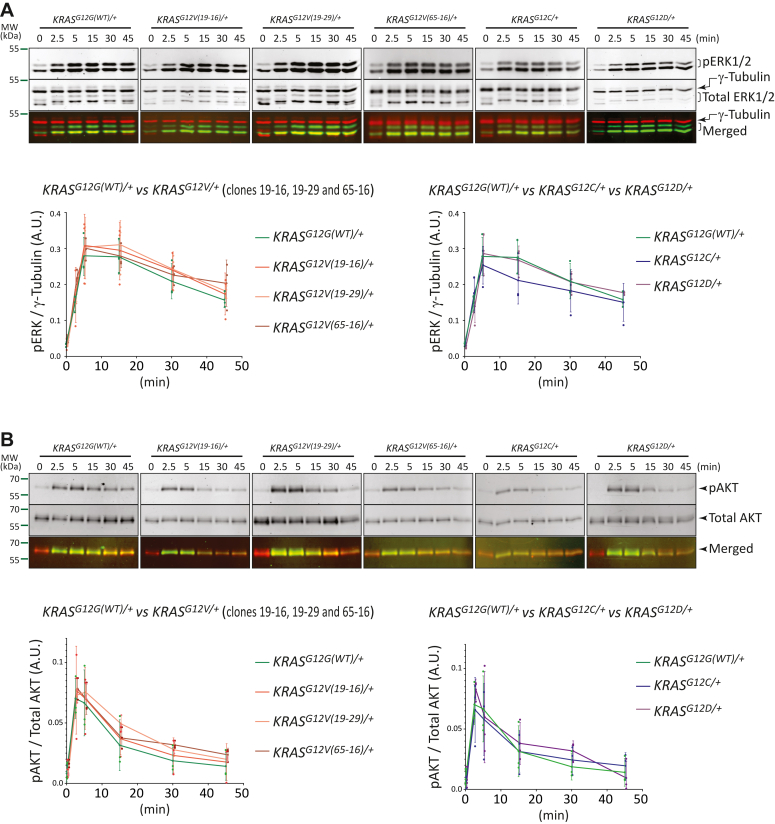
**ERK and AKT activation and attenuation profiles upon EGF treatment in the *KRAS***^***G12X/+***^**and *KRAS***^***G12G(WT)/+***^**cells.** Cells were serum-starved for 48 h before the EGF stimulation, as described in the Experimental procedures. ERK (*A*) and AKT (*B*) phosphorylation status were monitored for 2.5 min, 5 min, 15 min, 30 min, and 45 min after the EGF stimulation by Western blotting. The ratios of phosphorylated ERK1/2 (pERK) and the internal control γ-tubulin (*A*), or the ratio of phosphorylated AKT (pAKT) and the total AKT (*B*), were quantitated using the Odyssey imaging system (LI-COR). Three biological replicates, presented in Figs. S6 and S7, were analyzed to plot the graphs that show the mean and the SD values. In the left graph, the results of the *KRAS*^*G12V/+*^ (three clones, 19–16, 19–29 and 65–16) and the *KRAS*^*G12G(WT)/+*^ cells are plotted, and in the right graph, the results of the *KRAS*^*G12C/+*^ and the *KRAS*^*G12D/+*^ cells are plotted, where the *KRAS*^*G12G(WT)/+*^ data were included as a comparison. EGF, epidermal growth factor; pERK, phospho-ERK.

It is well accepted that oncogenic KRAS leads to the activation of the ERK and AKT signaling pathways (14). Interestingly, treatment of the oncogenic *KRAS*^*G12X/+*^ cells with EGF only led to a transient, but not a constitutive, activation of ERK and AKT as determined by their phosphorylation (Fig. 4, *A* and *B*). The attenuation of the phospho-ERK (pERK) and phospho-AKT (pAKT) signals occurred at a comparable rate between the oncogenic *KRAS*^*G12X/+*^ cells and the *KRAS*^*G12G(WT)/+*^ cells. Furthermore, asynchronously growing cells carrying the oncogenic *KRAS*^*G12X/+*^ mutations showed reduced pERK levels than the *KRAS*^*G12G(WT)/+*^ cells (Fig. S8). Collectively, cells expressing the oncogenic *KRAS*^*G12X/+*^ mutations did not exhibit a detectable increase in the ERK or AKT activity.

### *KRAS*^*G12V/+*^ cells show increased sensitivity to HU and a MEK inhibitor but are more resistant to a PI3K inhibitor compared to the *KRAS*^*G12G(WT)/+*^ cells

**Figure 5:**
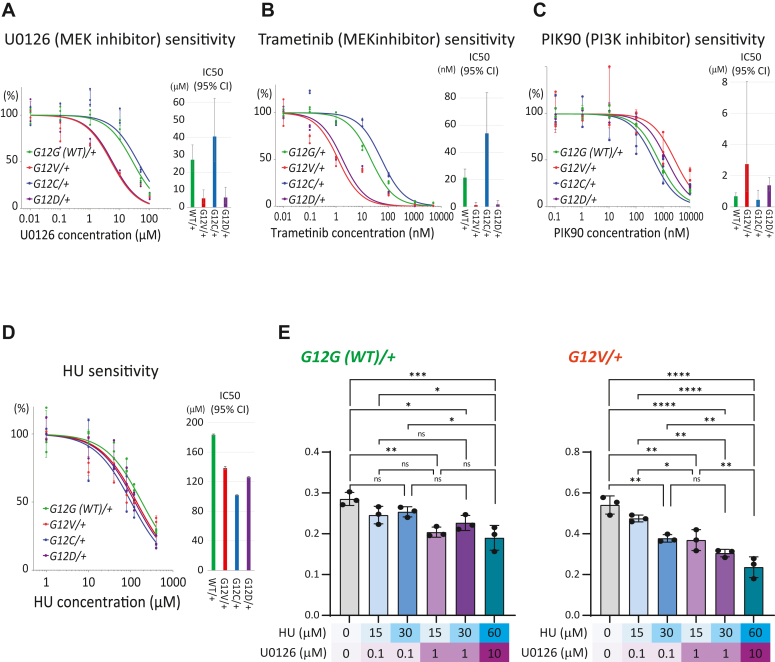
**Altered sensitivities of the *KRAS***^***G12X/+***^**and *KRAS***^***G12G(WT)/+***^**cells to U0126, trametinib, PIK-90 and HU.** Crystal violet-based cytotoxicity assays were conducted to monitor the sensitivities toward U0126 (*A*), trametinib (*B*), PIK-90 (*C*), HU (*D*) and the combination of U0126 and HU (*E*). For (*A–D*), three biological replicates were analyzed to generate the graphs that show the data points and SD values, together with the line representing the nonlinear fit of data produced by Prism (GraphPad). The best-fit IC50 values deduced from the data are summarized in the accompanying bar charts, where the error bars represent 95% CI (profile likelihood) produced by Prism (GraphPad). *A*, cells were exposed to the indicated concentrations of U0126 for 5 days before the fixation, staining, and cell lysis. *B*, cells were exposed to the indicated concentrations of trametinib for 5 days before the fixation, staining, and cell lysis. *C*, cells were exposed to the indicated concentrations of PIK-90 for 8 days before the fixation, staining, and cell lysis. *D*, cells were exposed to the indicated concentrations of HU for 7 days before the fixation, staining and cell lysis. *E*, the cells were subjected to six different combination treatments of HU and U0126, as shown in the graphs, for 6 days. Data for three biological replicates were obtained and analyzed with one-way ANOVA, followed by the Tukey *post hoc* test to compare the mean of each case with the mean of every other case. The *post hoc* test outcomes are shown in the diagrams except for all the neighboring cases, which show nonsignificant differences. All the statistical analyses were conducted using Prism (GraphPad). CI, confidence interval; HU, hydroxyurea.

We next explored whether one copy of the endogenous oncogenic *KRAS.G12X* mutation might alter drug sensitivity using inhibitors known to target Ras signaling pathways. Interestingly, the G12V and G12D mutant cells exhibited an increase in sensitivity toward two MEK inhibitors, U0126 and Trametinib, but were more resistant to PIK-90, a PI3K inhibitor, as compared to the *KRAS*^*G12G(WT)/+*^ cells (Fig. 5, *A–C*). Meanwhile, the G12C mutant cells behaved in a similar manner as the *KRAS*^*G12G(WT)/+*^ cells with these inhibitors. Sensitivities to Alpelisib, another PI3K inhibitor, were comparable among the G12V, G12D, and the WT KRAS cell lines, but the G12C mutant cells showed an increased resistance (Fig. S9). We also examined sensitivity against HU, a drug that interferes with DNA replication by inhibiting ribonucleotide reductase. The overexpression of oncogenic *HRAS.G12V* in human cells is reported to cause DNA hyper-replication and hyper-transcription, leading to replication stress (27, 28, 29). Hence, we suspected the sensitivity toward HU may be altered in the *KRAS*^*G12X/+*^ cells. Indeed, the oncogenic *KRAS*^*G12X/+*^ cells showed an increase in sensitivity toward HU, suggesting a possible defect in DNA replication control in these cells (Fig. 5*D*). Interestingly, HU and U0126 showed an additive inhibitory effect when applied simultaneously to the *KRAS*^*G12V/+*^ cells (Fig. 5*E*).

## Discussion

*KRAS* is among the most frequently mutated genes in human cancers. For over 40 years, extensive and continuous efforts have been made to understand the disease mechanisms initiated by mutant *KRAS* and to develop interventional therapeutics (30, 31). The recent development of RAS inhibitors marks a milestone and promises a brighter future for many cancer patients. It also redefines the current and future challenges, including improving inhibitor efficacies, mitigating acquired resistance, and developing new inhibitors that cover all oncogenic RAS isoforms (11, 32, 33). To address these issues, obtaining better insights into the mechanisms of oncogenic RAS signaling is essential. The set of isogenic cell lines that we developed in this study provides a new human cell culture model with defined genetic elements where the impact of one copy of an endogenous oncogenic *KRAS* mutation can be studied. As the cell lines harbor the inducible ER^T2^-Cre-ER^T2^ cassette, additional conditional gene editing is possible for future analyses (24).

The cell lines carrying the *KRAS.G12X* oncogenic mutations all showed an increase in the rate of cell proliferation compared to the *KRAS.G12G (WT)* control cell line, demonstrating that one copy of the endogenous *KRAS.G12X* oncogenic mutation is sufficient to induce this cancer relevant phenotype in hTERT RPE-1 cells. Interestingly, previous studies that targeted the endogenous *KRAS* allele using an adeno-associated viral vector to introduce the *KRAS.G13D* mutation in hTERT-HME1 cells (21) and the *KRAS.G12V* mutation into hTERT-IMEC cells (22) reported that the mutations did not increase cell proliferation or changes in cell morphology. Interestingly, the doubling time of hTERT-HME1 is reported to be about 30 to 40 h (21), much shorter than the doubling time of hTERT-RPE1 carrying *KRAS.WT* homozygously. An interesting possibility could be that the hTERT-HME1 might have lacked a G1/S regulation, which the hTERT RPE-1 may retain. Phenotypic differences in different host cell lineages may reflect the mechanism of oncogenic RAS signaling, and this point will be important to consider when planning future experiments.

Examination of their proliferation rate and cell cycle profile indicated that the G1 phase of the *KRAS*^*G12V/+*^ cells was substantially shorter compared to the *KRAS*^*G12G(WT)/+*^ cells. A similar phenotype was previously observed upon induction of *HRAS.G12V* expression in hTERT RPE1 cells (34). The shortened G1 phase is highly suggestive of a weakened restriction point, and the levels of the S807/S811 phosphorylation of retinoblastoma protein Rb (pRb-S807/S811), an indicator for the commitment into S phase (35), are increased in the asynchronously growing *KRAS*^*G12V/+*^ and *KRASG*^*12D/+*^ cells (Fig. S8). This altered pRb-S807/S811 status could be caused by an increase in cyclin D1 expression, which occurs upon oncogenic RAS overexpression (34, 36). However, we were not able to detect a significant change in Cyclin D1 expression in these cells (data not shown). Elucidating the mechanism underlying the shortened G1 phase is crucial to understanding how oncogenic *KRAS* increases the proliferation rate even when expressed from the endogenous locus.

An increase in cell motility, another cancer-related phenotype, was also observed in *KRAS*^*G12X/+*^ cells. Interestingly, the *KRAS*^*G12V/+*^ cells showed a reduced number of structures that stained for paxillin, suggesting that oncogenic KRAS signaling negatively impacts the focal adhesion complex assembly or maintenance, leading to increased cell motility. This observation is consistent with a previous study where deletion of the *KRAS.G13D* mutation from the endogenous locus of the human colorectal adenocarcinoma cell line, HCT116, results in an increase in paxillin structures (37). A reduction in paxillin structures can also be achieved by MEK inhibition in the parental HCT116 cells (37), indicating that the Raf-MEK-ERK signaling axis plays an important role in the oncogenic Ras-induced reduction in paxillin structures. Involvement of the Raf-MEK-ERK signaling axis was also suggested by the observations that sustained Raf activation induces paxillin phosphorylation at Ser126 and S130 (38), and active ERK is localized to and facilitates disassembly of focal adhesion complexes (39). However, the precise process of how active ERK facilitates focal adhesion complex disassembly still needs to be addressed, and we believe that our newly generated cell lines serve as an ideal experimental model.

Compared to the cell proliferation and motility phenotypes, the effect of one copy of the endogenous *KRAS.G12X* mutation on the ERK and AKT activation and attenuation profiles upon EGF treatment was not pronounced. ERK and AKT activation and attenuation profiles of the *KRAS*^*G12X/+*^ cells and the control *KRAS*^*G12G(WT)/+*^ cells were comparable. Furthermore, pERK levels in the asynchronously growing oncogenic *KRAS*^*G12X*^ cells were reduced compared to the *KRAS*^*G12G(WT)*^ cells (Fig. S8). These observations contrast with those in systems where oncogenic KRAS is overexpressed. However, they are in line with those in mouse embryonic fibroblasts (MEFs) isolated from the *KRAS.G12D* knock-in mouse model, where serum-stimulated MEK and AKT activation-inactivation profiles are similar between *KRAS.G12D* and control MEFs (40). Another MEF-based study, where mouse *HRAS*, *KRAS*, and *NRAS* have been replaced with a single human *KRAS* WT/oncogenic mutations, also demonstrated that the *KRAS* mutants produce ERK activation profiles comparable to the WT case by using a genetically encoded FRET-based ERK activity reporter for improved accuracy (41).

Considering that the oncogenic *KRAS.G12X* mutations caused increased cell proliferation and motility, it was surprising that the ERK and AKT activation status were comparable to the WT cells. One possibility could be that the oncogenic KRAS mutations may cause a subtle, undetectable change in ERK/AKT activation, which still can cause profound physiological consequences. Another possibility may be that the cellular localizations of ERK and AKT signaling pathway components may be affected, which have been relatively underexplored. It will be important to obtain the spatiotemporal activation status of ERK and AKT to fully understand the effect of endogenous oncogenic KRAS expression.

Besides ERK and AKT pathways, the RalA/B signaling axis has also been shown to play a key role in oncogenic Ras-mediated tumorigenesis (25, 42, 43, 44). Interestingly, we recently showed that the interaction kinetics between KRas and the RAS-associating domain of Rgl2, a RAS effector that activates RalA/B, is altered by the G12V mutation, whereas the binding kinetics between KRas and the RAS-binding domain of BRAF, that triggers the activation of the ERK pathway, is unaffected (45). It is important to examine whether the *KRAS*^*G12X/+*^ cell lines’ cancer-related phenotypes depend on the RalA/B function and how Rgl2 and RalA/B status are altered in these cell lines.

Having seen little effect on ERK and AKT signaling, it was interesting to observe the different sensitivities of the oncogenic *KRAS*^*G12X/+*^ and *KRAS*^*G12G(WT)/+*^ cells to the MEK inhibitors U0126 and trametinib and the PI3K inhibitors PIK-90. The *KRAS*^*G12V/+*^ and *KRAS*^*G12D/+*^ cells were more sensitive to the MEK inhibitors, potentially indicating that the cells were more reliant on (or “addicted to”) the ERK signaling pathway. On the other hand, the *KRAS*^*G12V/+*^ and *KRAS*^*G12D/+*^ cells were more resistant to PI3K inhibitors. Although the mechanisms behind these phenotypes are unclear, the results provide a rationale for investigating efforts to tailor the usage of MEK inhibitors for KRAS.G12V and KRAS.G12D–driven cancer treatments and highlight the importance of also considering the cytotoxicity of healthy cells when developing reagents against oncogenic KRAS signaling. Furthermore, it is intriguing that the *KRAS*^*G12C/+*^ cells responded to these inhibitors in a similar manner to the *KRAS*^*G12G(WT)/+*^ cells. The set of cell lines generated in this study can be a powerful tool to screen drug candidates that fulfill both high efficacy toward the specific oncogenic KRas mutation and minimum cytotoxicity toward healthy cells.

The increased HU sensitivity of the *KRAS*^*G12X/+*^ cells is consistent with the previous observation that oncogenic RAS expression causes replication stress (27, 28, 34, 46). Possible causes of the replication stress may include increased transcriptional activity (28), which may in turn be a consequence of hyperactivation of ERK signaling. Therefore, we examined whether simultaneous treatment with U0126 (MEK inhibitor) mitigates the HU sensitivity. Strikingly, U0126 exhibited an additive effect with HU on inhibition of growth of the *KRAS*^*G12V/+*^ cells, rather than mitigating the HU sensitivity. This finding may open a new approach to specifically target cells harboring oncogenic *KRAS* mutations.

In summary, we have generated a set of isogenic *KRAS*^*G12X/+*^ cell lines that will be highly valuable for further functional studies of oncogenic KRAS. We demonstrated that one copy of the endogenous *KRAS.G12X* oncogenic mutations causes cancer-relevant phenotypes, including an increase in cell proliferation and motility. This set of cell lines can also be a powerful tool to run pharmacological screens for the development of novel KRAS inhibitors with minimum cytotoxicity to healthy noncancerous cells.

## Experimental procedures

### Plasmid cloning

Gene editing at the *KRAS* locus was conducted as previously described (24). Two plasmids, pX330-kras_CRISPR and pKH-His-DA-Ap_kras_integration, were used to insert a LoxP cassette within the *KRAS* gene. The CRISPR plasmid, pX330-kras_CRISPR, is a derivative of pX330 (Addgene #42230) (47) where a *KRAS-*targeting guide RNA sequence, 5′ GTATTTCAGAGTTTCGTGAG 3′, has been inserted. The pKH-His-DA-Ap_*KRAS*_integration plasmid contains a DNA fragment encoding histidinol dehydrogenase under the phosphoglycerate kinase 1 promoter, which is flanked by a pair of LoxP sites. This LoxP cassette is sandwiched by *KRAS* homologous arms. The left (5′) arm spans position 9447 to 10,951 of chromosome 12, carrying an oncogenic G12V mutation in exon 2, and the right (3′) arm spans position 11,399 to 12,753 of chromosome 12. The pKH-His-DA-Ap_*KRAS*_integration plasmid was further mutated to carry other oncogenic mutations, G12C and G12D, as well as WT G12G.

### Cell culture, transfection, and drug treatment

To culture hTERT RPE-1 cells (ATCC, CRL-4000), Dulbecco's modified Eagle's medium (DMEM)/F-12 (Gibco #31331-028) containing 0.5% sodium bicarbonate, supplemented with 10% fetal bovine serum (Gibco #10500-064), 1% (v/v) Penicillin-Streptomycin (Gibco #15140-122) and 10 μg/ml Hygromycin B Gold (InvivoGen, #ant-hg-1) was routinely used. To culture hTERT RPE-1 ER-Cre-ER cells (24), the media were supplemented with puromycin at a final concentration of 7 μg/ml. Cells were cultured at 37 °C, 5% CO_2_. Transfection was conducted using Lipofectamine 3000 Reagent (Invitrogen, #L3000001) following the manufacturer’s instruction. To select clones with a LoxP cassette integration at *kras* gene locus, L-histidinol dihydrochloride (Sigma-Aldrich, #H6647) was added to the media at a final concentration of 1.35 to 1.5 mg/ml. For doxycycline treatment, doxycycline (Alfa Aesar, #J60422) was dissolved in water at 1 mg/ml and used at a final concentration of 1 μg/ml in the medium. For 4-OHT treatment, 4-OHT (Merck, #H6278) was dissolved in ethanol at 1 mM and used at a final concentration of 0.5 μM in the culturing medium.

For drug treatment experiments, following inhibitors were used: HU (Fluorochem #F043351-1G), dissolved in water at 20 mM, used at final concentrations of 1, 10, 40, 80, 120, and 400 μM, U0126 (Apollo Scientific #BIU1095), dissolved in dimethyl sulfoxide (DMSO) at 100 mM, used at final concentrations of 0.01, 0.1, 1, 10, 50, and 100 μM, trametinib (MedChemExpress #HY-10999A), dissolved in DMSO at 1 mM, used at final concentrations of 0.01, 0.1, 1, 10, 100, 1000, and 5000 nM, and PI3K inhibitors, PIK-90 (TOCRIS Bio-Techne #S1187) and Alpelisib (Insight Biotechnology CAS 1217486-61-7), dissolved in DMSO at 1 mM and 10 mM respectively, were used at final concentrations of 0.1, 1, 10, 100, 1000, and 10,000 nM (PIK-90) and 0.05, 0.5, 5, 50, 500, and 5000 nM (Alpelisib).

### PCR based genotyping

For genome extraction, about 1 × 10^6^ cells were collected and were lysed in 400 μl of lysis solution (100 mM NaCl, 25 mM EDTA, 20 mM Tris–Cl (pH 8.0), 0.5% SDS, 0.5% 2-Mercaptoethanol, 0.2 mg/ml Proteinase K (Thermo Fisher Scientific EO0491)). The sample was incubated at 55 °C for 18 to 24 h. On the following day, 450 μl of phenol:chloroform:isoamyl alcohol 25:24:1 was added, and the sample was vigorously mixed. It was centrifuged, and then the upper aqueous was transferred to a fresh tube containing 180 μl of 7.5 M ammonium acetate and 750 μl of 100% ethanol to precipitate the genome. The sample was centrifuged, and the pellet was air-dried and dissolved in 50 μl of Tris buffer (Thermo Fisher Scientific, #K0721).

To amplify the *KRAS* locus, a pair of primers Kras_intron1_F-2022(5′ AGCCACCGTGCCCGGCTCACTTGC 3′)and KRAS__R_Nov22 (5′ GGAGGTCTTTGAGATTAAATAAATCCTCATCTGCTTG 3′) were used. A typical PCR reaction mix was prepared in 25 μl reaction scale by assembling the following components; 1 μl of extracted genome DNA (11–35 ng/μl), 0.75 μl of each primer (10 μM), 0.75 μl of dNTPs (10 mM each), 0.5 μl of KAPA HiFi DNA polymerase (Roche, #KK2101), 5 μl of 5 × KAPA HiFi buffer (Roche, #KK2101) and 16.75 μl H_2_O. The PCR condition was set as follows; 3 min denaturation at 95 °C, 35 cycles of “20 s 98 °C, 15 s 65 °C, 90 s 72 °C” and 5 min 72 °C. The amplified fragments were sequenced using the primer Alu_genome_checkF (5′ TCATTACGATACACGTCTGCAGTCAACTGG 3′).

### Preparation of complementary DNA

To examine the coding sequence of the edited *KRAS* gene locus and to confirm the heterozygous expression of the WT and the edited *KRAS* gene loci, total RNA was isolated using Direct-zol RNA miniprep kit (Zymo research, #R2051 and #R2050-1-50) following the manufacturer’s instruction. The mRNA quality was examined by Agilent Bioanalyzer to confirm the RNA integrity number to be 9.5 to 10. About 5 μg of the purified mRNA was used to generate complementary DNA using the Goscript kit (Promega #A5001). The KRAS coding region was amplified using a pair of primers Kras_Exon1-F (5′ CCGCCATTTCGGACTGGGAGCGAGCGC 3′) and Kras_3′UTR_S (5′ CTGCATGCACCAAAAGCCCCAAGACAGAAATCTTAGG 3′), and the sequencing was conducted using the primers Kras_3′UTR_S and AlugenomecheckF.

### Preparation of cell extracts of growth-factor stimulated cells

*KRAS.G12G*, *KRAS.G12C*, *KRAS.G12D*, and *KRAS.G12V* hTERT RPE-1 cells were grown to 70 to 80% confluent in 6-cm cell culture dishes (TPP, #93060). The cells were then serum-starved in the DMEM/F-12 media for 48 h. The starved cells were growth-factor stimulated with fresh DMEM media containing 100 ng/ml EGF Human (#A63411-500 (Antibodies.com)), and time-course samples were collected at 0, 2.5, 5, 15, 30, and 45 min after the EGF stimulation. To collect cell extracts, the media were removed and the cells were washed once with 3 ml of PBS and lysed in 120 μl of the modified 3× Laemmli sample buffer that contains 8M urea (192 mM Tris–Cl (pH 6.8), 4.8% SDS (w/v), 24% glycerol (v/v), 1.8 M β-mercaptoethanol, 0.0048% bromophenol blue, and 8M urea). Before loading samples into the 12% SDS-PAGE gel, they were heat-denatured at 95 °C for 5 min.

### Western blotting

Proteins were resolved by SDS-PAGE and transferred to 0.2 μm nitrocellulose membranes (Bio-Rad, #1620112). The membranes were blocked overnight at room temperature with a blocking buffer (LI-COR Intercept Blocking Buffer TBS, #927-60001). The following primary antibodies were used: anti-AKT (Cell Signaling Technology, 40D4 Mouse mAb #2920, dilution 1/2000), anti-pAKT (Cell Signaling Technology, rabbit Antibody #9271, dilution 1/1000),anti-ERK (Thermo Fisher Scientific: Invitrogen, mouse antibody #10221703, dilution 1/500), anti-pERK (Cell Signaling Technology, rabbit mAb #4370, dilution 1/2000), anti-KRas antibody (OriGene, mouse monoclonal #CF801672, dilution 1/1000), anti-Ras.G12V antibody (Cell signaling technology, rabbit monoclonal #14412, dilution 1/800), anti-γ-tubulin antibody (Sigma-Aldrich, mouse monoclonal, #T6557, 1/5000 dilution) and anti-pRb-Ser807/S811 antibody (Cell Signaling Technology, rabbit mAb #8516, 1/1000 dilution). To detect the primary antibodies, goat anti-mouse IgG antibody (LI-Cor, #926-68020) and IRDye 800CW goat anti-Rabbit IgG antibody were used as a secondary antibody. Membrane images were acquired by Odyssey Infrared Imaging System (LI-COR Biosciences). The protein levels were quantified using Image Studio software (LI-COR Biosciences). Three biological replicates were conducted and analyzed by Prism software (https://www.graphpad.com, GraphPad).

### Cell proliferation assays

Cells were initially cultured in a 9-cm plate (CytoOne Dish, Starlab #CC7682-3394) until they reached 90% confluence before trypsinization and resuspension in fresh media. Subsequently, the cells were seeded into 6-well plates (Sarstedt, #83.3920). At this stage, we generated a few parallel sets of samples where the cell densities ranged from 2 × 10^3^ to 4 × 10^3^ cells per well. After 4 days of incubation, cell numbers were counted, and we chose the sample set where the wells contained 5 × 10^3^ to 1 × 10^4^ cells on average and used this set for further proliferation analysis. This was to mitigate influences from the initial lag time. The cell proliferation status was assessed using a haemocytometer until the cell density reached 1 × 10^5^ cells/ml and then using CellDrop (DeNovix) as previously described (24). Briefly, cell numbers were counted every 24 to 48 h for up to the total incubation time of 264 h (11 days) by trypsinizing the cells and staining them with 0.4% trypan blue solution (Sigma-Aldrich T8154).

### Cell growth inhibition assays

Cell growth inhibition assays were conducted essentially following the previously reported protocol (48). Briefly, cells were grown in 6-well plates (Sarstedt, #83.3920) till 60 to 80% confluency, washed once with PBS, and were stained with crystal violet staining solution (0.5% crystal violet powder (Sigma-Aldrich C6158), 20% methanol) for 10 min at room temperature. The staining solution was discarded, and the cells were gently but thoroughly rinsed with water. The stained cells were air-dried before they were lysed with the lysis buffer (0.1 M sodium citrate and 50% ethanol, pH 4.2). The samples were incubated for 30 min at room temperature, gently shaking to ensure complete lysis of the cells. The generated samples were examined by a spectrophotometer at 590 nm absorbance. Three biological replicates were analyzed by Prism software (GraphPad).

### Analysis of DNA content by flow cytometry

Cells were trypsinized, harvested by gentle centrifugation, and washed with PBS. They were fixed by resuspending in 70% cold ethanol, and the samples were stored at −20 °C until the measurement. The stored samples were washed with PBS three times and were incubated in the staining solution (PBS containing 5 mM of EDTA, 20 μg/ml of propidium iodide (Sigma-Aldrich #81845) and 200 μg/ml RNAse A (New England Biolabs #T3018-1)) for 4 h on ice. The resultant samples were measured with FACSCanto II (BD Biosciences), and the data analysis was done using BD FACSDiva (BD Biosciences).

### Quantitative live cell imaging

The modes of cell migration were quantitatively measured using the Livecyte kinetic cytometer 2 (Phasefocus) which allows label-free imaging. Cells in 1 ml media were seeded in a 24-well plate coated with Collagen Type 0 (Jellagen, #Jel1018) 48 h before imaging. Cell images were taken every 20 min intervals using a PLN 10 ×/NA 0.25 objective for 10 h at 37 °C in an atmosphere supplemented with 5% CO_2_. Acquired images were analyzedfor instantaneous velocity, migration speed, and cell displacement using Livecyte Analysis software (https://www.phasefocus.com/livecyte, Phasefocus).

### Immunofluorescence microscopy

Cells were cultured on collagen-coated coverslips (BioCoat Collagen I 22 mm Round #1, Falcon #CF905) and prior to the fixation, cells were rinsed with PBS. The cells were fixed with freshly prepared 3.7% paraformaldehyde for 5 min at room temperature. The cells were then incubated in 0.2% Triton X-100 for 2 min to increase cell permeability and were quenched with 1 mg/ml NaBH_4_ for 4 min, followed by 1 h of incubation at room temperature in 0.1 M glycine solution. The prepared samples were blocked in the blocking medium (PBS supplemented with 4% fetal bovine serum (Gibco #10500-064) and 1% bovine serum albumin (BSA) (Thermo Fisher Scientific #BPE9702-100)) overnight. The samples were then incubated with appropriate primary antibodies diluted in PBS supplemented with 1% (w/v) BSA for 1 h at 37 °C. The following primary antibodies were used in this study: anti-paxillin antibody (BD Biosciences #610052, mouse monoclonal, dilution 1/500) and anti-Rgl2 antibody (Novus Biologicals, mouse monoclonal, #H00005863-M02, dilution 1/500). The cells were rinsed with PBS for three times and incubated with the following reagents prepared in PBS supplemented with 1% (w/v) BSA; CF594-conjugated goat anti-mouse IgG1(γ1) (Biotium, #20249), at a final concentration of 2 μg/ml, 4′,6-diamidine-2′-phenylindole dihydrochloride (DAPI) (Merck #10236276001) at a final concentration of 1 μg/ml, and Phalloidin-iFluor 488 Reagent (Abcam, ab176753), 1/100 dilution. The samples were incubated for 40 min at room temperature. The samples were finally rinsed with PBS three times, once with Milli-Q water and mounted on a slide using Vectashield mounting medium (Vector Laboratories, H-1000-10). Cell images were acquired using a 2D array scanning laser confocal microscope (Infinity 3, Visitech) using a 60×/1.4 Plan Apo objective lens (Nikon). Each image comprised 35 serial images with 0.3 μm intervals along the Z-axis to span the full thickness of the cell.

### Quantification of paxillin structures

**Supplemental Figure S5:**
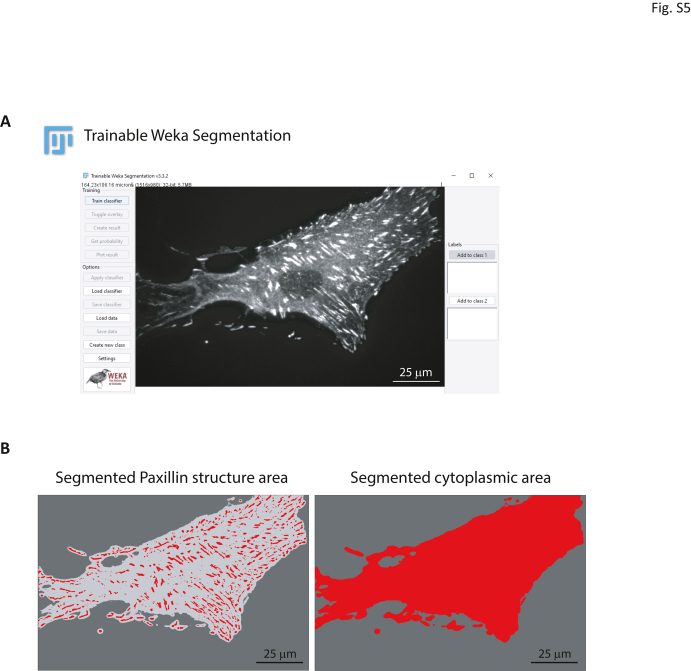


**Supplemental Figure S8:**
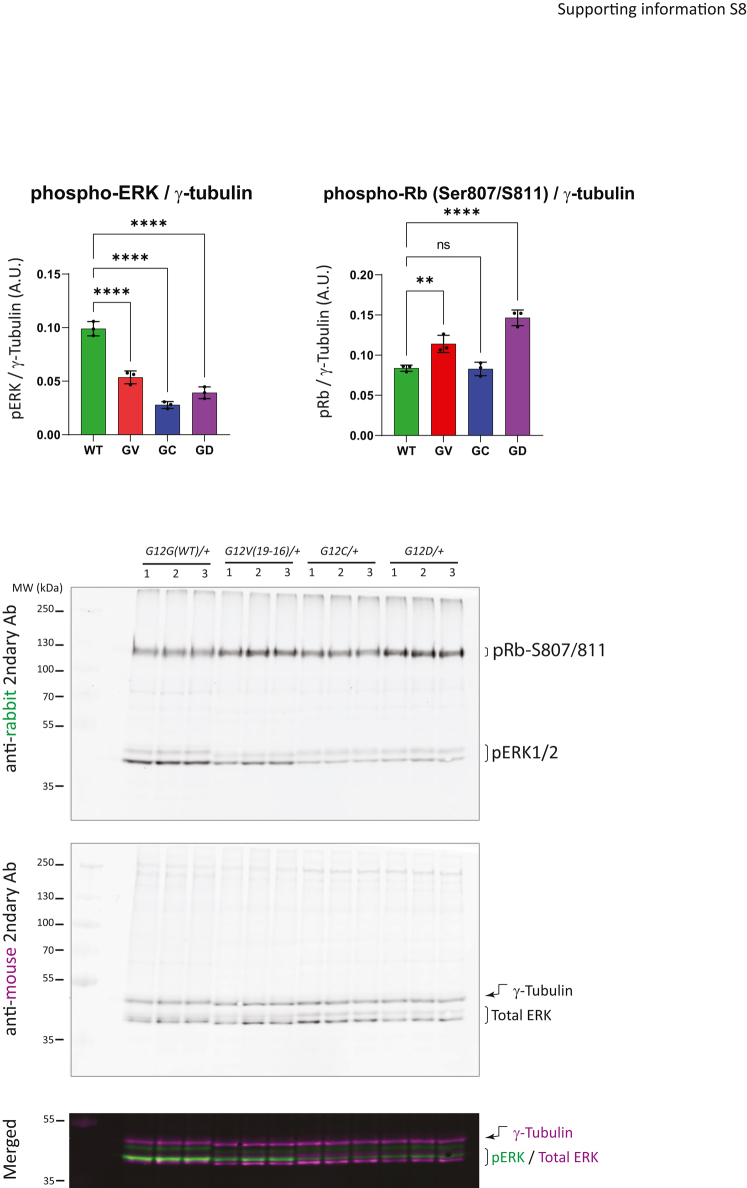


In order to appreciate the change in the number of the paxillin structure in a cell, the ratio of the area of paxillin structures and the area of the cytoplasm was quantified using the Fiji Plug-in “Trainable Weka Segmentation” (49, 50). Firstly, all 35 Z-slices of each image were stacked and summed to form one image. To this image, “Trainable Weka Segmentation” was applied as stated in the Fig. S4 and two binary images were generated; one which represents the area of paxillin signals and the other which represents the area of cytoplasm (including the paxillin area). Using these images, the paxillin:cytoplasm ratio was calculated as described in Fig. S5. For each biological replicate, 20 images were quantified. The data from three biological replicates were analyzed with nested *t* test using Prism (GraphPad).

## Data availability

The datasets supporting the conclusions of this article are included within the article and its additional file.

## Supporting information

This article contains supporting information.

## Conflict of interest

The authors declare that they have no conflicts of interest with the contents of this article.

## Figures and Tables

**Supplemental Figure S3, A and B:**
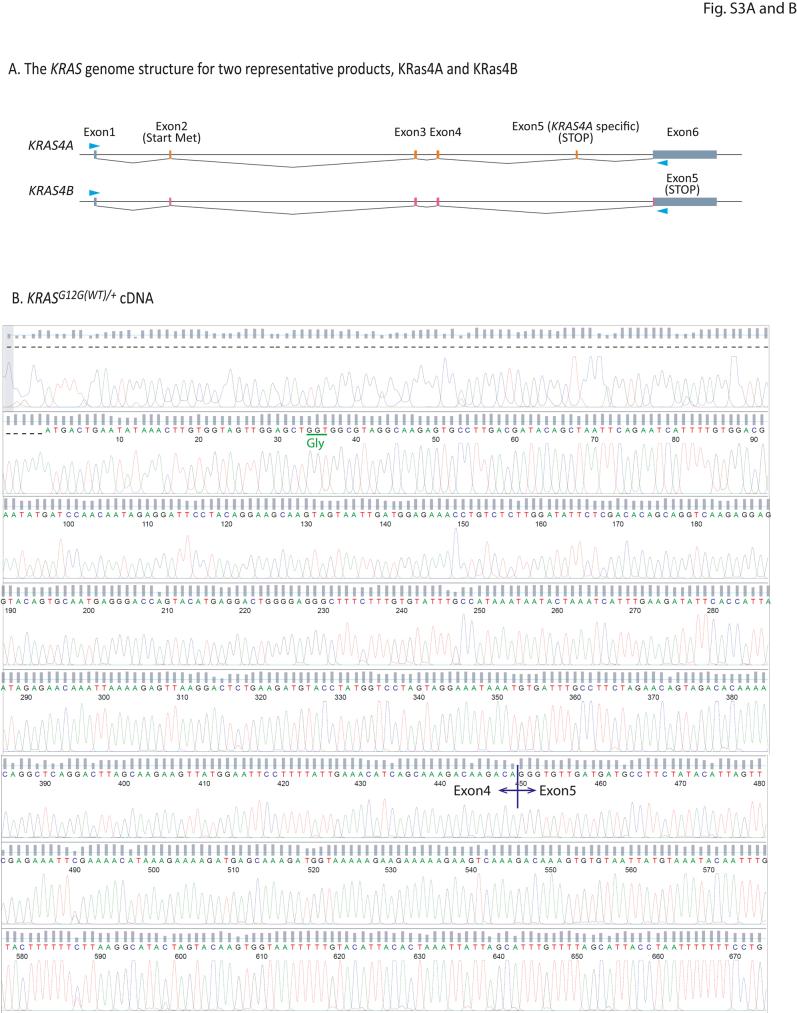


**Supplemental Figure S3C:**
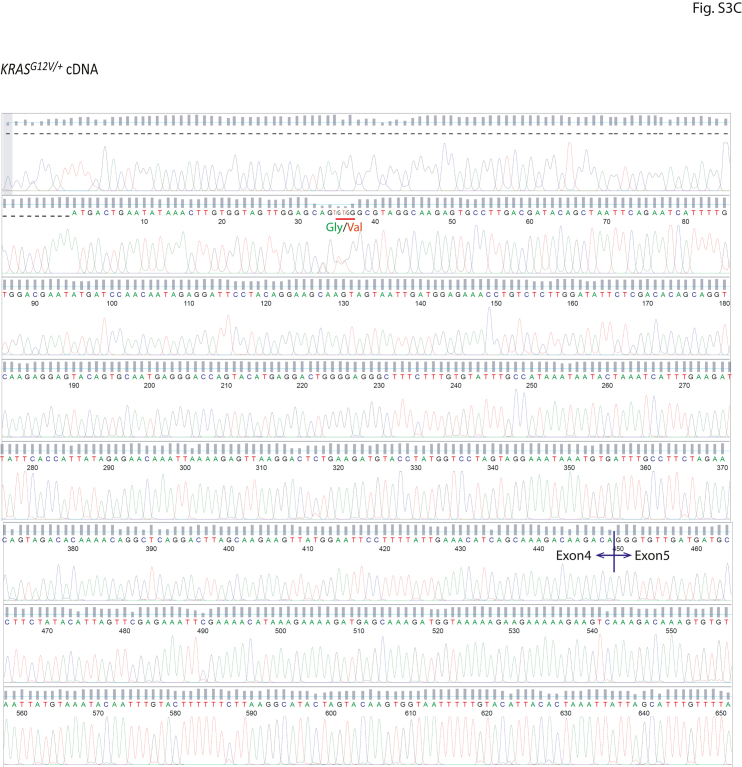


**Supplemental Figure S3D:**
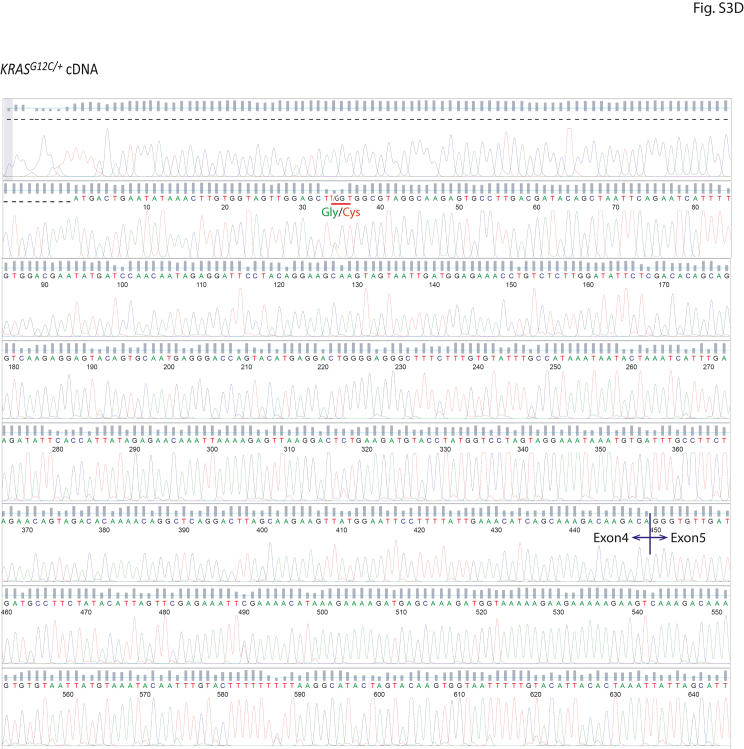


**Supplemental Figure S3E:**
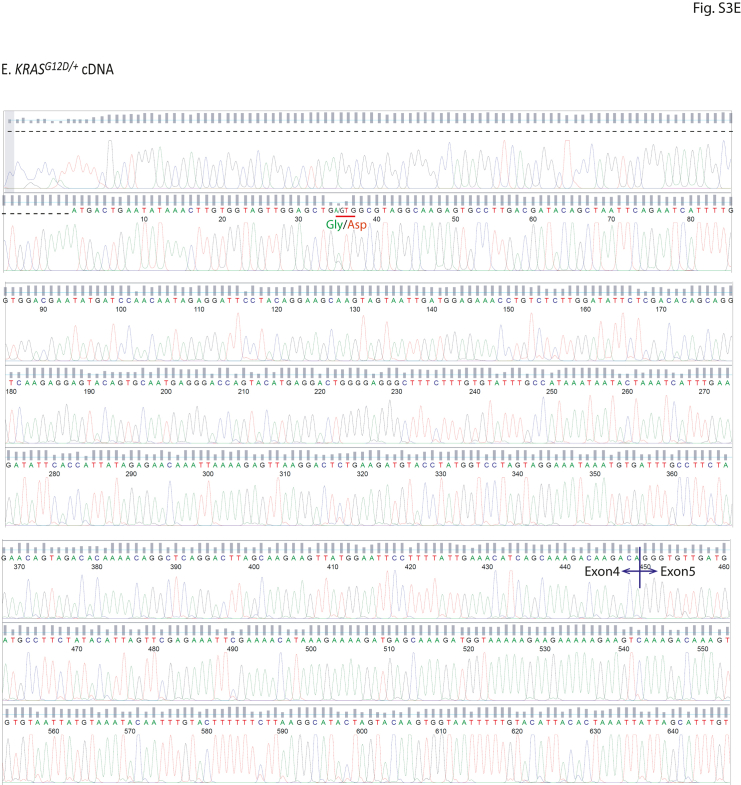


**Supplemental Figure S6:**
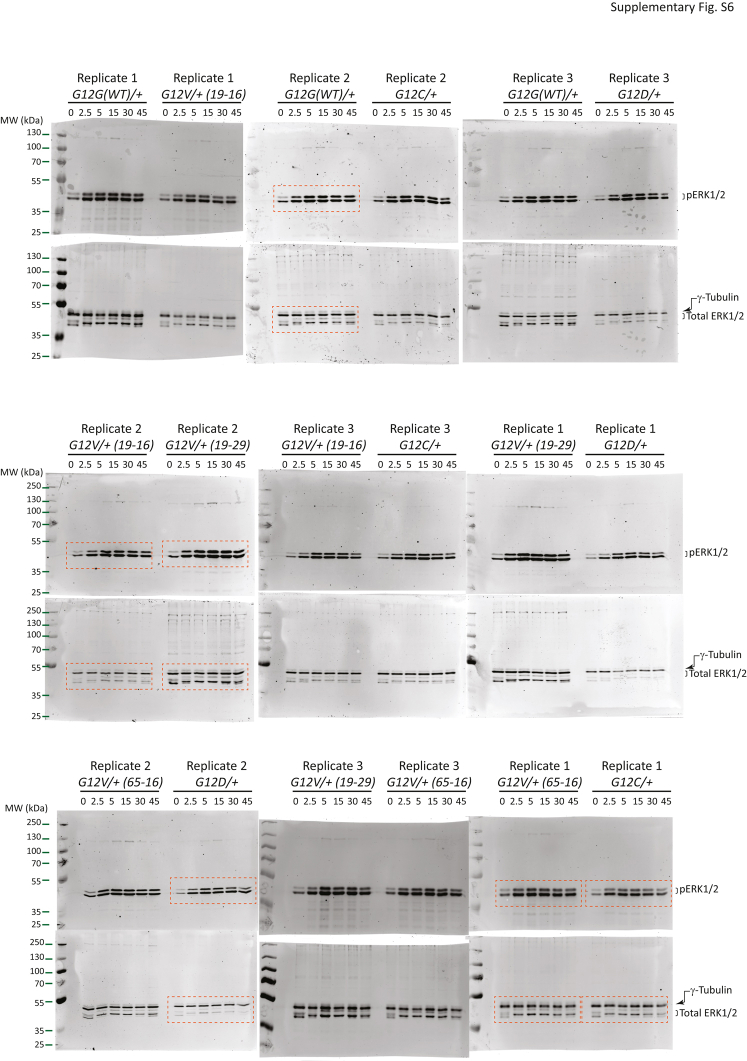


**Supplemental Figure S7:**
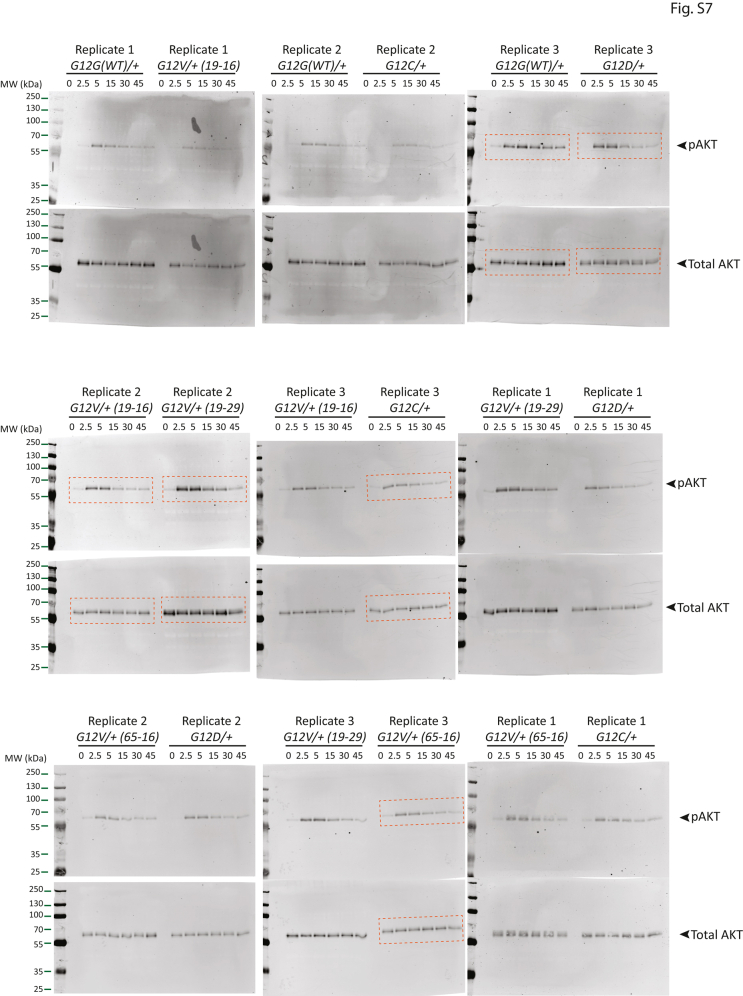


**Supplemental Figure S9:**