# Supplementary material for: Endogenous oncogenic KRAS expression increases cell proliferation and motility in near-diploid hTERT RPE-1 cells
Source: J Biol Chem. 2024 May 23;300(6):107409. doi: 10.1016/j.jbc.2024.107409 (PMC11234024; doi:10.1016/j.jbc.2024.107409)
Supplement: Supplemental Figure S1–S9 [file mmc1.docx]

**Supporting Information**

**Supporting information S1. hTERT RPE-1 cells carry a heterozygous c.30_35dup mutation at the *KRAS* gene allele** (A) The PCR-based genotyping of the *KRAS* exon2. Using a pair of primers, AlugenomecheckF and Kras_R, the *KRAS* exon 2 of both of the chromosomes of hTERT RPE-1 cells were amplified. The location of the c.30_35dup insertion mutation is indicated as an orange star in exon2. The agarose gel image shows successful amplification of the expected gene fragment, indicated by an arrow. (B) The PCR product shown in (A) was sequenced using the primer AlugenomechekF to reveal that one of the chromosomes carries the c.30_35dup insertion mutation. The six nucleotides corresponding to the c.30_35 are orange-underlined, and the inserted duplicated AGCTGG nucleotides are highlighted in orange. The encoded amino acids of the presented region are shown in grey letters below the nucleotide sequences. The c.30_35dup mutation causes the duplication of Ala11_Gly12, leading to the additional Ala_Gly at positions 13 and 14 (shown in orange letters). The Gly12 and Gly13 oncogenic hotspot amino acids are shown in magenta.

**Supporting information S2. The LHL cassette does not repress the expression of KRas.G12V protein.** (A) Cell extracts were prepared from four independent *KRAS^G12V/+^* clones (clone 19-16, 19-29, 19-32 and 65-16), which were cultured either in the absence (-) or presence (+) of doxycyclin and 4-OHT that excise the LHL cassette. As a reference, the parental hTERT RPE-1 ER^T2^-Cre-ER^T2^ cell extracts were also prepared. The expression of KRas.G12V was detected by the G12V-specific antibody, whereas the total KRas expression was probed by the KRas-specific antibody. As an internal control, γ-tubulin levels were also detected. The G12V expression levels were comparable between the samples treated or untreated with doxycyclin and 4-OHT, indicating that the LHL cassette does not interfere with the transcription or translation of the KRas.G12V. It was also confirmed that the total KRas expression levels are comparable between the parental cell line and the generated *KRAS^G12V/+^* cell lines, indicating that the KRas expression levels are kept to the endogenous level in the generated cell lines. (B) Original Western blotting membranes for the data presented in (A).

**Supporting information S3. Sequence analysis of the cDNAs of the generated cell lines.** (A) Schematic representations of two representative *KRAS* transcripts, *KRAS4A* (KRAS-201, ENST00000256078.10) and *KRAS4B* (KRAS-202, ENST00000311936.8). These two transcripts share exons 1, 2, 3, and 4, but exon 5 of *KRAS4A* is within intron 4 of *KRAS4B*. The locations of the primers, Kras_Exon1-F and Kras_3’UTR_S, used for the amplification of the cDNA, are indicated as blue arrowheads. (B) - (E) The sequencing chromatograms of the cDNAs prepared from the *KRAS^G12G(WT)/+^* cells (B), *KRAS^G12V/+^* cells (C), *KRAS^G12C/+^* cells (D) and *KRAS^G12D/+^* cells (E). The codon that encodes the amino acid position 12 is underlined. The junction of exon 4 and exon 5 is indicated. The sequence signal of *KRAS4B* exon 5 was the only signal that was detected.

**Supporting information S4. The original flow cytometry data used for Figure 2.** (A) Three biological replicates of *KRAS^G12G(WT)/+^* cells. (B)-(D) Three biological replicates of *KRAS^G12V/+^* cells (B), *KRAS^G12C/+^* cells (C) and *KRAS^G12D/+^* cells (D). Cell populations in sub-G1, G1, S and G2 were set as P3, P4, P5 and P6, respectively. 10000 data points (cells) were counted and analysed.

**Supporting information S5. The quantitation protocol to deduce the ratio of the area of the paxillin structures and the area of the cytoplasm.** (A) Fiji trainable Weka segmentation Plug-in was used for the quantitation. First, the classifier was trained to recognise the paxillin structure and the cytoplasm using an example image to establish a classifier condition. The classifier condition was then applied to the images to be analysed for pixel-based segmentation; twenty images were analysed for each replicate of the *KRAS^G12G(WT)/+^* and *KRAS^G12V/+^* image set. (B) Binary images representing the paxillin structure and the cytoplasm were generated. For each image, the “IntDen (integrated Density)” was measured. The area ratio of the Paxillin structure and the cytoplasm was deduced by calculating “IntDen(Paxillin structure) / IntDen(cytoplasm)”. For each image, one “IntDen(Paxillin structure) / IntDen(cytoplasm)” value was obtained. As each replicate contains 20 images, 20 values of “IntDen(Paxillin structure) / IntDen(cytoplasm)” were obtained for each replicate. Three replicates were analysed for both the *KRAS^G12G(WT)/+^* and *KRAS^G12V/+^* image sets. The total data size was 20 data points for three replicates. The data was analysed by the nested t-test (GraphPad Prism) (Fig. 3C).

**Supporting information S6. Original Western blotting membranes for ERK activation-attenuation profiles of three biological replicates presented in Fig. 4A.** Enclosure squares of the orange dotted line indicate the images used in Fig. 4A.

**Supporting information S7. Original Western blotting membranes for AKT activation-attenuation profiles of three biological replicates presented in Fig. 4B.** Enclosure squares of the orange dotted line indicate the images used in Fig. 4B.

**Supporting information S8. The phosphorylation status of ERK and Rb in the asynchronously growing *KRAS^G12G(WT)/+^* and *KRAS^G12X/+^* cells.** Asynchronously growing cells were lysed, and the cell extracts were prepared as described in the Experimental Procedures. ERK and Rb phosphorylation status were monitored by Western blotting. The ratios of phosphorylated ERK1/2 (pERK) and the internal control γ-tubulin (left panel), or the ratios of phosphorylated Rb (pRb-S807/S811) and the internal control γ-tubulin (right panel), were quantitated using the Odyssey imaging system (Li-COR). Three biological replicates were analysed to plot the graphs that show the data measurement points and the SD values. Data for three biological replicates were analysed with one-way ANOVA, followed by the Tukey post-hoc test to compare the mean of each case with the mean of other case. The post-hoc test outcomes are shown in the diagrams. All the statistical analyses were conducted using Prism (GraphPad). *****P*<0.0001, ***P*=0.0065, ns *P*=0.9984.

**Supporting information S9. Sensitivities of the *KRAS^G12X/+^* and *KRAS^G12G(WT)/+^* cells to Alpelisib (PI3K inhibitor)** Crystal violet-based cytotoxicity assays were conducted to monitor the sensitivities towards Alpelisib. Three biological replicates were analysed to generate the graphs that show the data points and SD values, together with the line representing the non-linear fit of data produced by Prism (GraphPad). The best-fit IC50 values deduced from the data are summarised in the accompanying bar-charts, where the error bars represent 95% CI (profile likelihood) produced by Prism (GraphPad). Cells were exposed to the indicated concentrations of Alpelisib for 8 days before the fixation, staining and cell lysis.
